# Differential evolution algorithm based photonic structure design: numerical and experimental verification of subwavelength *λ*/5 focusing of light

**DOI:** 10.1038/srep30871

**Published:** 2016-08-01

**Authors:** E. Bor, M. Turduev, H. Kurt

**Affiliations:** 1 Department of Electrical and Electronics Engineering, TOBB University of Economics and Technology, Ankara, Turkey; 2 Department of Electrical and Electronics Engineering, TED University, Ankara, Turkey

## Abstract

Photonic structure designs based on optimization algorithms provide superior properties compared to those using intuition-based approaches. In the present study, we numerically and experimentally demonstrate subwavelength focusing of light using wavelength scale absorption-free dielectric scattering objects embedded in an air background. An optimization algorithm based on differential evolution integrated into the finite-difference time-domain method was applied to determine the locations of each circular dielectric object with a constant radius and refractive index. The multiobjective cost function defined inside the algorithm ensures strong focusing of light with low intensity side lobes. The temporal and spectral responses of the designed compact photonic structure provided a beam spot size in air with a full width at half maximum value of 0.19λ, where λ is the wavelength of light. The experiments were carried out in the microwave region to verify numerical findings, and very good agreement between the two approaches was found. The subwavelength light focusing is associated with a strong interference effect due to nonuniformly arranged scatterers and an irregular index gradient. Improving the focusing capability of optical elements by surpassing the diffraction limit of light is of paramount importance in optical imaging, lithography, data storage, and strong light-matter interaction.

Light interaction with an absorption-free medium can experience reflection, refraction, diffraction, and coherent or incoherent scattering^1^. Conventional optical materials and structures manipulate light propagation with a limited capability. For example, total-internal reflection-based light guidance in fiber optic cable or integrated optical waveguides is only one type of light confinement mechanism deployed. It was only recently that multidimensional periodic variation of the refractive index with a strong contrast ratio provided an energy band gap concept similar to the electronic band gap occurring due to the interaction of electron movement with the periodic potentials of atoms in crystalline solids. The forbidden energy interval is closely linked with Bragg scattering. As a result, tailoring light propagation with a photonic band gap mechanism has resulted in photonic crystal waveguides and photonic crystal fibers^2^.

Photonic crystals (PC) are periodically designed artificial structures that manipulate the flow of light, and their periodicity can be one, two, or even three dimensional^2^. PCs have many remarkable properties that make it indispensable to control the photon movement using structures such as waveguides^3^, cavities^4^, bio-chemical sensors^5^, flat lenses^6^, and low-threshold lasers^7^. Photons may also be trapped or emitted using a single defect^8^.

Even though there are various light manipulation scenarios, one of the widely targeted problems is to improve focusing characteristics of photonic structures. The wave nature of light dictates the limitation to focus light below a certain spot size smaller than half of the wavelength of light. A common way of focusing light requires either mirrors or lenses with curved surfaces and constant refractive indices^1^. Reflection and refraction are responsible for bringing light into focus in these cases. Meanwhile, one can use a diffraction mechanism to focus incident light by deploying diffractive optical elements^9^. For the latter case, there is no need for curved surfaces. Even though the two approaches have different structural configurations, they share the property of being homogenous, i.e., the refractive index is kept constant in definite regions.

Light focusing can also be accomplished by taking advantage of an inhomogeneous medium that provides an index gradient, and the approximated version of the continuous index gradient can be achieved by means of PCs^10^. Graded index (GRIN) PCs designed via dispersion engineering or the effective medium theory have contributed to realize novel light manipulation capabilities. The main restriction for this approach is that one has to mimic and follow the given input graded index profile. Methods other than using GRIN PCs can also be used to focus light. These methods include using metal nanoslit lenses^11^ and structural layers that consist of air holes^12^. Moreover, a genetic algorithm was applied to remove some of the rods of a PC structure for designing a spot size converter^13^.

The ability to control light at subwavelength dimensions has become an important research interest in recent years. The importance of subwavelength focusing covers different applications for imaging^14^,^15^,^16^, nanofabrication^15^,^16^, manipulation^15^,^16^,^17^,^18^,^19^,^20^, and nanofocusing^21^. By means of tightly focused light beams, we can manipulate particles and objects with smaller dimensions. Moreover, subwavelength imaging may help to view the details of cells or very small biological particles such as DNA, viruses, and proteins. A dielectric PC slab has been used to achieve canalization for focusing light at the subwavelength scale with a full width at half maximum (FWHM) value of 0.059*λ*_0_^22^. In that study, the main purpose is to transport the focal point to long distances. However, the focusing property of the structure for short distances is not considered and there occurs strong reflection at the interface between air and structure. Hypergratings are demonstrated to obtain FWHM values between *λ*_0_/20 and *λ*_0_/50^23^. In ref. 24 planar anisotropic metamaterial with zone plates is introduced for the purpose of subwavelength focusing of light where the calculated FWHM value of focal point is equal to *λ*_0_/10. However, metamaterial structures suffer from high material absorption losses due to the imaginary part of the metalic material permittivity. In the case of the focusing by metamaterial structures, absorption also affects the focal point resolution that limits the subwavelength focusing performance of the structure. Additionally, off-axis focusing characteristic in subwavelength scale by nanoscale slits with metamaterial slab is reported where the FWHM value is equal to 22 nm at the operating wavelength of 365 nm. That corresponds to a FWHM value to be approximately 0.060*λ*_0_^25^. In this case, because of the material losses inside the structure, the intensity of focused light is very low compared to the source light. The phase compensation mechanism is proposed for focusing the plane wave to a spot by constructive interference in a metamaterial with FWHM value of 0.14*λ*_0_^26^. Also, an optical hyperlens system is proposed that has resolution capabilities better than *λ*_0_/2^27^. An important question can be asked: what would happen if an optimization algorithm is utilized to design all dielectric structure to obtain subwavelength focusing of light?

Designing different and complex photonic structures based on theoretical knowledge and an intuition approach is limited. For this reason, a variety of optimization algorithms have been used to design efficient photonic integrated structures. A genetic algorithm was used to design a spot size converter, lenses, a demultiplex coupler and a large photonic band gap structure^13^,^28^,^29^,^30^. The same algorithm was also used to generate different orders of Hermite–Gauss profiles^31^. In addition, topology optimization was applied to obtain a photonic band gap and design a waveguide bend, mode converter, and subwavelength focusing structure^32^,^33^,^34^,^35^. Another optimization technique called particle swarm optimization was used to obtain directional emissions at the exit of the PC waveguide and engineer slow light parameters of a line defect waveguide^36^. An algorithm based on convex optimization was applied to design a wavelength demultiplexer^37^. Also, a nonlinear search algorithm was recently used to design a beam-splitter^38^, a gravitational search algorithm to design a nanophotonic cavity^39^, and the direct binary search algorithm to design optical diodes^40^,^41^.

In the present work, we introduce a differential evolution (DE) algorithm to design a subwavelength focusing PC structure composed of all dielectric scattering elements. As we demonstrate later, the transverse cross section of the input beam was shaped by the designed photonic structure. We achieved strong focusing of the beam with carefully controlled side lobe intensity levels. Microwave experiments were performed to verify the numerical results.

In the following parts of the paper, we outline the implementation of the DE algorithm to design a lens for subwavelength focusing. The distance between the dielectric rods was optimized to achieve strong light focusing. The results show that the proposed optimization strategy can be used to design a subwavelength focusing PC with desired beam properties via defining multiobjectives. The proposed approach possesses some advantages. For example, all dielectric materials are utilized. There are no metallic or exotic materials deployed in the design. As a result, the subwavelength focusing structure is inherently free from absorption loss and can work for a broad wavelength interval.

## Definition of the problem and design approach of algorithm

The main aim of the proposed work was to find a desired photonic structure that resulted in subwavelength focusing of the illuminating wave. The relevant parameters for the most critical characteristics of a focusing apparatus are the spatial size and the cross-sectional profile. These geometric properties are important for different applications such as beam size shaping, high resolution imaging, and the high efficiency of light coupling. The size of the focal spot is characterized by the FWHM value, and for subwavelength focusing it should be smaller than λ/2 in air. While considering the strong light focusing below λ/2, one has to pay attention to the emergence of side lobes, which should not carry a large amount of the beam’s energy.

If a large amount of the energy is shared with the side lobes, then an undesired radiation pattern may emerge after propagating a certain distance, and the interaction of light with the medium at the focal spot would become weaker. When the side lobes of the focal point are reduced, the FWHM of the main lobe (focal spot) is usually increased. There is a trade-off to be solved in the present work. In order to consider not only a small FWHM but also fairly suppressed side lobes compared to the main lobe, multiobjective optimization can be utilized as a possible solution.

**Figure 1:**
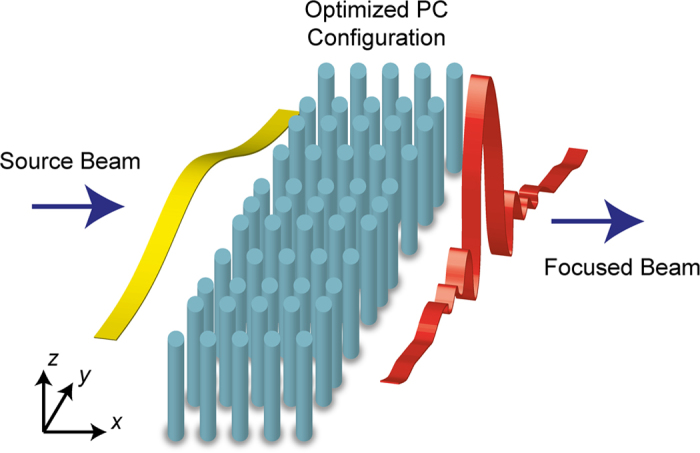
Schematic representation of the strong light focusing problem. Dielectric PC rods with the constant refractive index *n*_*r*_ = 3.13 are placed in an air background with the refractive index *n*_*air*_ = 1.0.

Figure 1 pictorially outlines the design approach implemented in the DE algorithm. The intensity profiles of the incident and transformed beams are figuratively presented. Our purpose is to use the DE algorithm to optimize the beam shaping structure.

**Figure 2:**
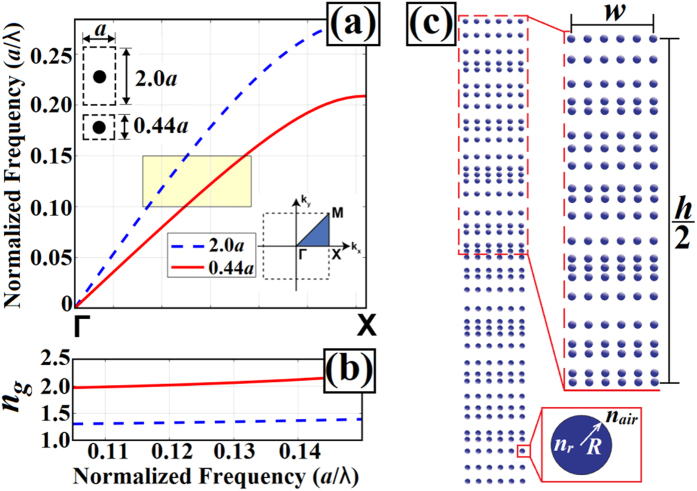
(**a**) Dispersion curves of rectangular cells with different dimensions. (**b**) Group index curves for the two extreme cases corresponding to the smallest and largest cells. (**c**) The DE generated two-dimensional photonic crystals.

In this study, the filling ratio/effective index of the PC structure was modulated and optimized using the DE algorithm to obtain a strong focusing effect. Refractive index modulation can be achieved by engineering the PC parameters, such as gradually changing the material index, filling factor and/or lattice period^42^,^43^,^44^. Moreover, index modulation can also be accomplished by introducing different duty cycles that locally modulates the effective index of refraction in subwavelength gratings^45^,^46^ and inhomogeneous grating structures with lensing effect^47^. In the current work, we utilized the last method, where size optimization of the PC unit cells was performed while keeping the material type and rod radii the same. The adjustment of the size of the PC unit cells resulted in a modulation of the effective index of refraction, which allowed the structure to manipulate phase fronts of the propagating wave. The radii of the PC rods were fixed at 0.20*a*, and their refractive indices were kept at *n* = 3.13. (The PC rods were considered alumina rods for the microwave experiment). The lateral dimension of the unit cells ranges from Δ*y* = 0.44*a* to Δ*y* = 2.0*a* according to the optimization. The lattice constant is represented by *a*. Moreover, the distance between rods along the propagation *x*-direction is fixed to 1*a* (*i.e*., the modulation of the effective index performed along only the transverse *y*-direction). Corresponding variations of cell sizes in transverse y-direction are depicted in the Fig. 2a as an inset. It should be noted that there is a restriction on the placement of dielectric rods. The maximum distance between the centers of PC rods in the same column is 2.0*a*. Since the diameter of each rod is 0.40*a*, the minimum distance between adjacent rods in y-direction should be greater than 0.40*a* so they do not touch each other. For this reason, the minimum distance was fixed at 0.44*a*. Using these structural parameters, we calculated the dispersion diagrams of PC unit cells with maximum and minimum lateral sizes by exploiting the plane wave expansion (PWE) method in the frequency domain^48^. The dispersion diagram is shown in Fig. 2a. The related bands move to higher frequencies while the unit cell size increases (the lateral dimension increases and the longitudinal dimension is kept constant). We should note that throughout all numerical analyses, only transverse magnetic (TM) polarization was employed, where the concerned nonzero electric and magnetic field components are E_*z*_, H_*x*_, and H_*y*_, respectively. Additionally, we designed the photonic structure operating in the first band.

The group indices of each band (*n*_*g*_) were extracted using the slope information of the relevant curves presented in Fig. 2a, and the result is illustrated in Fig. 2b. For the longer wavelengths in the first band, the curves are closely spaced and deviate in a linear manner with respect to the normalized frequency. The calculated group indices within the normalized frequencies *a*/*λ* = 0.10 to 0.14 cover values between 1.40 and 2.20. It can be concluded that different values of Δ*y* provide specific *n*_*g*_ values within the spectral window.

After determining the initial conditions of the approach, the DE optimization algorithm was incorporated into the design for subwavelength focusing. DE is a metaheuristic method to provide a sufficiently good candidate solution to multiobjective optimization problems. Since DE does not search the complete design space, the obtained solution is not the best solution but one of the efficient solutions that satisfy the intended conditions for the optimization problem under study. Cost function, which is part of DE and necessary for convergence, should be designed carefully in order to find a good solution^49^. In our designs, we applied the “best/1/bin” method of DE. At the beginning of the optimization, the algorithm randomly generated a population of a certain number of PC structures, called individuals. Each individual, with its own cost value, was made of a fixed number of parameters (i.e., the distances between PC rods). Individuals with better cost values were selected to generate the population of the next iteration. The algorithm ends when the defined cost value of any individual satisfies the desired termination condition or when the maximum number of iterations which is defined at the beginning of the algorithm is reached.

As explained above, the DE was applied to optimize the distances between dielectric rods (i.e., the lateral size of the unit cell in a column). Since an objective of the proposed work was also to generate a compact and feasible PC structure, one should define the structural restrictions. Hence, the maximum distance between the centers of rods in the same column is 2.0*a*, and the minimum distance is 0.44*a*. To obtain the focal point at the optical axis, we used mirror symmetry with respect to the *x*-axis. As a result, the algorithm was run to generate only half of the structure, and that half was duplicated symmetrically to obtain the complete configuration. Moreover, the cost function was defined to minimize the FWHM value and suppress the intensity level of the maximum of side lobes (MSL) to stay under 20% of the normalized arbitrary value of the intensity profile at the focal point. We set the desired value for FWHM close to zero since the lowest possible FWHM value was targeted for subwavelength focusing. At the beginning of the algorithm, the number of columns and the normalized frequency were defined by the user. Furthermore, to see the effect of the longitudinal size of the optimized PC structure on focusing ability, we applied DE for a different number of columns as the values varied from 5*a* to 10*a* for each design frequency. One more important parameter is the distance between the focal point and back surface of the PC structure. In our optimization, we calculated the FWHM values at least 0.32*a* distance away from the end of the structure. The cost function is mathematically defined in equation (1):


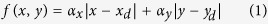


where *x*_*d*_ and *y*_*d*_ are the targeted FWHM and desired MSL values, respectively. Variables *α*_*x*_ and *α*_*y*_ are the weighting factors that balance the effect of FWHM and MSL values to the value of the cost function.

## Numerical Results

Numerical modeling of the optimized PC lens and its time-domain analysis were realized using the finite-difference time-domain (FDTD) method^50^,^51^. In all numerical simulations, the computational domain was restricted to a 2D spatial domain and the third dimension was taken to be uniform. To eliminate undesired back reflections, the boundaries of the computational domain were surrounded by perfectly matched layers^52^. A continuous source with a Gaussian profile was utilized to calculate the spatial intensity distribution of the focusing effect of the optimized PC lens.

**Figure 3:**
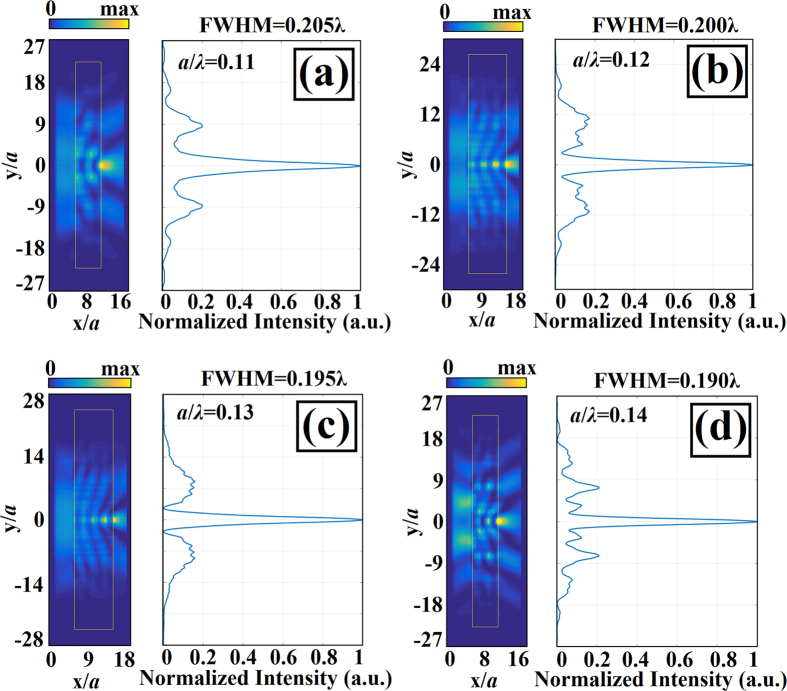
Different photonic structures generated by implementing the algorithm. (**a**) Steady state intensity profiles at *a*/*λ* = 0.11 for the structure with 6 columns. Similar plots (**b**) at *a*/*λ* = 0.12 for 10 columns, (**c**) at *a*/*λ* = 0.13 for 10 columns, and (**d**) at *a*/*λ* = 0.14 for 6 columns.

The algorithm was applied to design different structures for different normalized frequency values. For each normalized frequency, different numbers of columns were chosen to design photonic structures. The schematic view of the optimized PC structure is presented in Fig. 2c. An optimized PC lens with a better cost value was selected from among the structures designed with the same normalized frequency value. Therefore, the number of columns in selected PCs was varied independently from the normalized frequency values. In Fig. 3, intensity distributions and cross sections of the field at the focal point are given. Related figures of PCs with normalized frequency values *a*/*λ* = 0.11, 0.12, 0.13, and 0.14 are shown in Fig. 3a–d, respectively.

As the results show in Fig. 3, subwavelength focusing down to FWHM = 0.19*λ* was achieved. The other FWHM values were below 0.50*λ* as well. Also, it is clear that the side lobes were symmetrical with respect to the *x*-axis. The FWHM and MSL values were calculated, and the results of the structures satisfy the desired conditions of DE. In general, we obtained around *λ*/5 FWHM values and reduced maximum side lobe levels to under 20% of the maximum value of 1.0 for each PC. Moreover, the spot-size conversion ratio of each selected structure was calculated. The results are summarized in Table 1.

We should point out that while subwavelength focusing was achieved with the suppressed side lobes, we strictly pushed the level of the side lobes to stay below a certain value. Moreover, the calculated values of the energy fraction of main lobe are given in Table 1. As it is seen from the results, around 40% of overall energy is concentrated within the main lobe of the focused beam.

**Figure 4:**
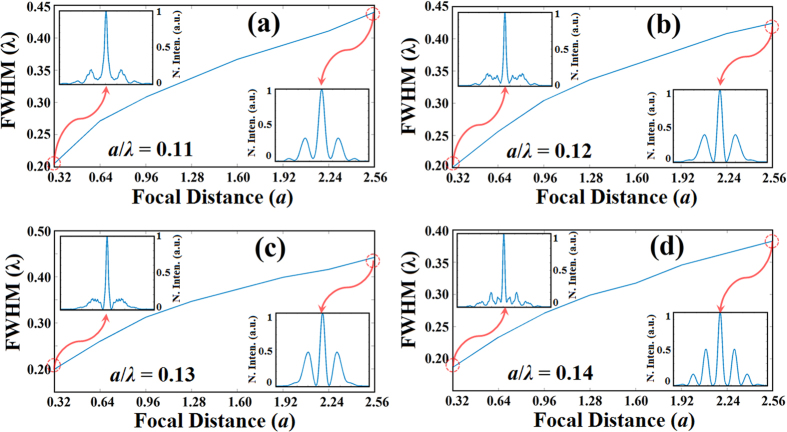
Plots of numerically calculated full width half maximum (FWHM) values depending on focal distance for four different structures that designed at the normalized frequency values equal to (**a**) *a*/*λ* = 0.11, (**b**) *a*/*λ* = 0.12, (**c**) *a*/*λ* = 0.13, and (**d**) *a*/*λ* = 0.14. For each plot, transverse cross-sectional profiles of intensity at the focal distance values of 0.32*a* and 2.56*a* are given as insets in the top-left and bottom-right corners, respectively.

For the four different designed structures which are presented in Fig. 3, FWHM values at different focal distances are numerically calculated and FWHM dependence on focal distances is given as plots in Fig. 4. As can be seen from the figure, focal distances are varied from 0.32a to the 2.56a away from the structure with 0.32a steps for all four different structures. Furthermore, to show the transverse cross-sectional intensity profile variation in terms of focal distance values of 0.32a and 2.56, we included insets for each designed structure in Fig. 4a–d. As it is seen from Fig. 4, the variations of the FWHM values with increasing of focal distance values are nearly linear and FWHM values stay under the diffraction limit for greater values of focal distances increasing from 0.32a to 2.56a. Even though the level of side lobes increase as focal distance increases, MSL values stay suppressed under the 50% of the normalized intensity at each focal distance value.

One can argue that two physical mechanisms govern the strong focusing of light. The first one is the rather irregularly placed dielectric rods providing an index gradient. Although there is irregular spacing between dielectric locations, as shown in Fig. 2c, the overall density of dielectric material is higher at the center of the structure and decreases toward the edges. The indication of the index gradient can be seen in the phase front changes apparent in the intensity profiles, as shown in Fig. 3.

The other mechanism is based on the interference of light at structure places that have a wide distance between rods. These places guide light and promote multibeam interference. An electric field exists at the exit of each waveguide channel, and the interference of waves emanating from the N-channel provides a total scalar electric field in the form of the following expression:


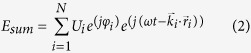


In equation (2), the amplitude and phase of the field at the exit side of the waveguide are represented by 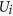 and 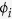, respectively. Also *ω* and *N* represent radial frequency of propagating wave and number of channels, respectively. Time harmonic dependency is assumed in the equation. The phase term contributing to the interference effect for each channel is determined by the dot product of the wave vector, 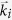, and the position vector, 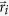. The intensity profile is determined by 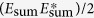 where 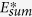 is complex conjugate of the total electric field. Obtaining a closed form expression for the intensity profile without any approximation or assumption for the above equation is not straightforward. However, we can point out that the parameters that play roles for the intensity profile are amplitude profiles, relative phase terms, and the number of interfered waveguides. A large *N* yields a narrower peak at the central part of the structure with a higher intensity value. In other words, as *N* increases, the central lobe concentrates more tightly due to the increased number of waveguide channels. The presence of the side lobes is also an indication of a multibeam interference effect. As a result of these two phenomena, strong focusing of the beam occurs at the outside of the structure. Since we do not know the phase difference between each fictitious channel and amplitudes of the wave at the exit of the structure, we do not attempt to derive an intensity distribution profile at the back side of the structure. We solely share that equation in order to discuss the importance of the nonuniformly distributed amplitudes and phase variation at the end of the structure that plays role for the focusing effect.

## Experimental Results

**Figure 5:**
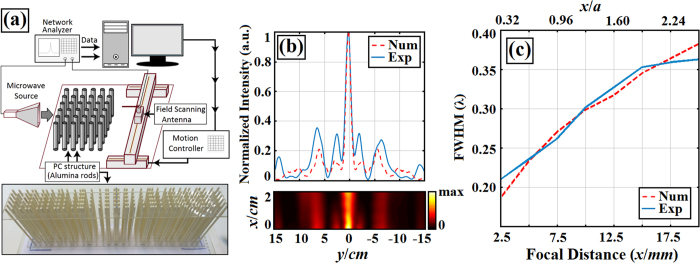
(**a**) Schematic and photographic view of the experimental setup used to investigate the optimized PC lens (optimized at *a*/*λ* = 0.14 normalized frequency) at the microwave region. (**b**) The measured electric field and its transverse cross-sectional profile at 5.42 GHz with a 0.21*λ* FWHM value. (**c**) Plot of experimentally measured and numerically calculated FWHM values depending on focal distances.

To verify the numerical results, we performed experimental realization at the microwave region. We used dielectric alumina rods to generate the PC lens, as in the numerical calculations. Figure 5a shows the schematics and photographic illustration of the experimental setup. A vector network analyzer called Agilent E5071C ENA was used to generate a wave source and record the intensity field of the focused wave. To excite the structure and measure the steady-state intensity distribution, the horn and monopole antennas were employed, respectively. The operating frequency of the antennas ranges between 4 and 6 GHz.

We tried to carefully match the numerical and experimental excitation conditions by placing the source at the entrance of the PC lens in both cases. Additionally, microwave absorbers were placed around the structure to reduce possible back reflections. A TM polarized wave was launched to the designed PC lens. Using the monopole antenna, the intensity distribution was obtained by measuring the focused field intensity at the air background focal plane of the structure. The monopole antenna was placed parallel to the rods (perpendicular to the x-y plane), and the tip of the antenna was placed at the half-height of the alumina rods. The studied structure is composed of cylindrical Alumina rods with a refractive index of *n *= 3.13 and a radii of rods are equal to *r *= 1.55 mm. The regarding lattice constant is then set to *a *= 7.75 mm. The steady state intensity field at the back focal plane was measured by moving the monopole antenna 300 mm in the y-direction and 25 mm in the x-direction with spatial steps of 2.5 mm. The upper part of Fig. 5b shows the superimposition of the cross-sectional intensity profile at the operating frequency of 5.42 GHz obtained from experiments (solid line) and that obtained from 2D numerical calculations (dashed line). As can be seen from the plot, a good match between the simulated models and experimental realization was achieved. The lower part of Fig. 5b presents the electric intensity field profile of the focused wave at the same operating frequency. The measured FWHM value from the experimental data is 0.21λ, and the MSL stays below the normalized intensity value of 0.30. If one compares the measured and calculated FWHM values, the mismatch between them is negligible (i.e., the difference between the calculated value and the measured one is 0.02λ). Both numerical and experimental FWHM values at different focal distances are shown in Fig. 5c. In experiments, distance of 20 mm, along the x-axis is scanned. FWHM values are measured at distances from 2.5 mm to 20 mm with 2.5 mm steps. As it is seen, the experimental FWHM values at different focal lengths stay under the diffraction limit and agree well with the numerical values of FWHM. Moreover, the experimentally measured values of the energy fraction of main lobe of the focused beam are equal to 30.5% of overall energy at the focal point.

Since our lattice constant is *a* = 7.75 mm and operating normalized frequency is *a/λ* = 0.14 we can find that our operating wavelength equal to *λ* = 55.35 mm. As can be seen from Fig. 5(c) the subwavelength focusing with FWHM equal to 0.21*λ* emerges at focal distance equal to ΔF = 2.5 mm or ΔF = 0.0452*λ* and FWHM increases until 0.36*λ* while ΔF increases to ΔF = 0.3613*λ*. Therefore, strongly focused beam occurs between focal distances of 0.0452*λ* and 0.3613*λ*. In this case, one can deduce that the designed structure works as a subwavelength focusing lens in the near-field, i.e. light localization within the ΔF < *λ*/2 limit. It is widely known that near-field strong focusing of light is useful in various areas such as for bio-imaging, near-field lithography, optical memory storage, light harvesting, and spectral signal enhancing^53^. Moreover, near-field focusing properties are important to super-resolution applications including field localization, fabrication^54^,^55^, characterization, sensing and imaging^56^, near-field scanning optical microscopy^57^ and near field infrared spectrometry^58^.

**Figure 6:**
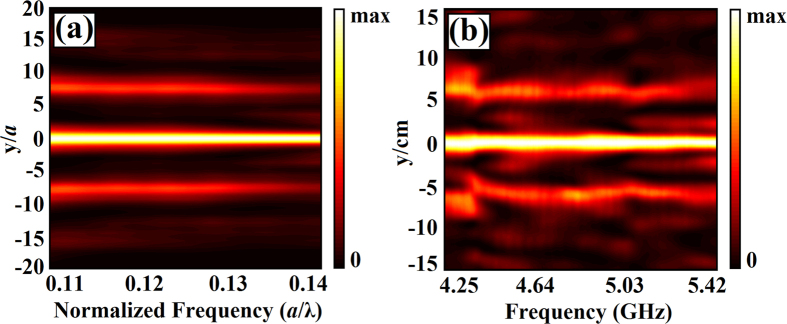
(**a**) Numerical and (**b**) experimental plot of the transverse cross section intensity map at the back focal plane of the PC lens at a distance of x = 2.5 mm from the structure for different operating frequencies ranging from *a*/*λ* = 0.11 to 0.14 and 4.25 to 5.42 GHz, respectively.

To show the effectiveness of the designed PC lens as a subwavelength focusing device within a wide range of operating frequency, we also performed experimental steps for the frequency range of 4.25 to 5.42 GHz, which can be presented as normalized frequencies of a/λ = 0.11 to 0.14. Figure 6a,b show the transmitted transverse cross-sectional intensity maps, plotted as a function of the incident wave carrier frequency of numerical and experimental results. Figure 6a shows the subwavelength focusing effect with FWHM values of 0.205λ, 0.200λ, 0.195λ, and 0.190λ at defined normalized frequencies of a/λ = 0.11, 0.12, 0.13, and 0.14, respectively. In Fig. 6b, the map of experimental cross sections shows that frequencies of 4.25, 4.64, 5.03, and 5.42 GHz provided the expected strong focusing effect with subwavelength FWHM values of 0.234λ, 0.230λ, 0.225λ, and 0.210λ, respectively. Even though the main features appearing in both cases are similar, there is a discrepancy between them due to the fact that microwave experiment with finite horn antenna apertures was conducted with finite step size (spatial resolution). In addition to that, it is fairly difficult to bring the receiver antenna very close to the end surface of the structure. As a result, we see some deviation between the experimental and numerical results presented in Fig. 6.

**Figure 7:**
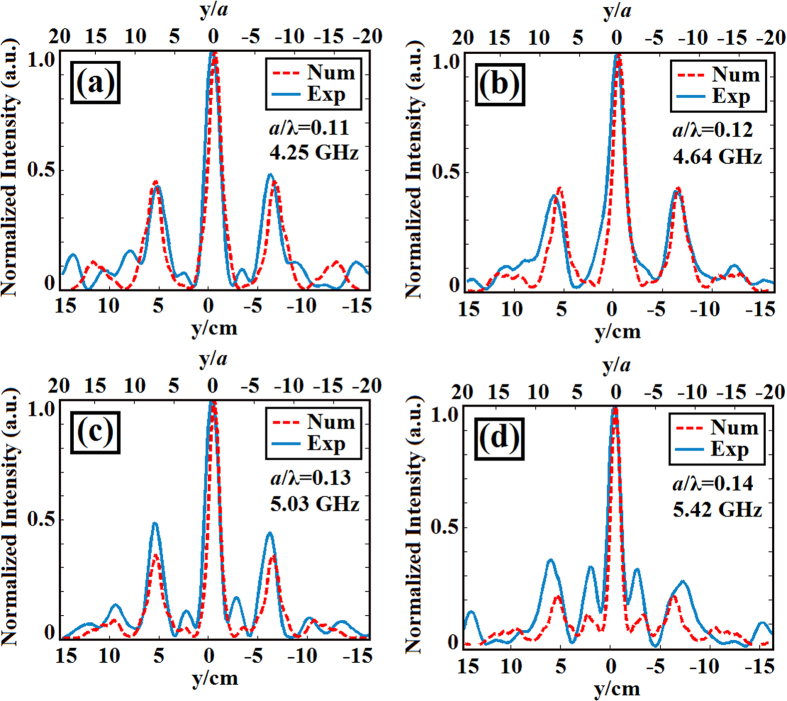
Transverse cross-sectional intensity profiles of the experimental (solid lines) and FDTD (dashed lines) results taken at (**a**) 4.25 GHz (*a*/*λ* = 0.11), (**b**) 4.64 GHz (*a*/*λ* = 0.12), (**c**) 5.03 GHz (*a*/*λ* = 0.13) and (**d**) 5.42 GHz (*a*/*λ* = 0.14) with FWHM values of 0.234*λ* (FDTD: 0.205*λ*), 0.230*λ* (FDTD: 0.200*λ*), 0.225*λ* (FDTD: 0.195*λ*) and 0.210*λ* (FDTD: 0.190*λ*), respectively.

When we inspect the transverse intensity profiles of light at the four operating frequencies, we see subwavelength focusing of the beam as summarized in Fig. 7. The numerical and experimental results are consistent with each other. Moreover, it is clear from these figures that the focused waves have side lobe levels staying under 50% of the maximum intensity value. This result is also proof of the broadband nature of the designed photonic structure. The subwavelength focusing of light is achieved at more than one frequency.

## Conclusion

In this paper, we proposed an optimization algorithm known as differential evolution (DE) to design a photonic medium made of lossless dielectric circular elements. The aim was to find a photonic structure that provides strong focusing of light. The algorithm was integrated into a finite-difference time-domain method, and multi-objectives such as narrow full width at half maximum and low-intensity side lobes were defined in the cost function. To the best of the authors’ knowledge, the optimization algorithm based on DE was utilized for the subwavelength focusing for the first time. FWHM values less than 0.25λ in air with low-intensity side lobes were demonstrated both numerically and experimentally. The subwavelength focusing of light is linked with the interference effect and irregular index gradient. Strong focusing of light may improve the resolution of optical imaging and lithography and increase the data storage capacity of optical media. The proposed DE algorithm can be applied to design other photonic structures for additional light manipulation techniques.

## Additional Information

**How to cite this article**: Bor, E. *et al*. Differential evolution algorithm based photonic structure design: numerical and experimental verification of subwavelength λ/5 focusing of light. *Sci. Rep*. **6**, 30871; doi: 10.1038/srep30871 (2016).

## Figures and Tables

**Table 1:** Summary of results obtained from four different designed PCs.

Normalized Frequency (*a*/*λ*)	Number of Columns	FWHM	Maximum Side Lobe Level	Spot-Size Conversion Ratio	Fraction of Energy of Main Lobe
0.11	6	0.205λ	0.199	9.34	56.69%
0.12	10	0.200λ	0.168	12.08	41.35%
0.13	10	0.195λ	0.148	12.62	43.87%
0.14	6	0.190λ	0.199	12.95	39.79%
